# Diverse *Staphylococcus aureus* lineages in a tertiary hospital in North Central Nigeria: emergence of CC1-ST772-MRSA, CC8-ST789-MRSA, and ST152-MRSA

**DOI:** 10.3389/fmicb.2026.1908867

**Published:** 2026-10-09

**Authors:** Rasaki Adewole Raheem, Charles Nwabuisi, Abayomi Fadeyi, Bashirat Ayobola Olanipekun, Ademola Ibraheem Hamzat, Tomiwa Olumide Adesoji, Taofiq Adewale Sulayman, Alexander Mellmann, Frieder Schaumburg, Adebayo Osagie Shittu

**Affiliations:** 1 Institute of Medical Microbiology, University of Münster, Münster, Germany; 2 Department of Medical Microbiology and Parasitology, University of Ilorin Teaching Hospital, Ilorin, Nigeria; 3 Department of Medical Microbiology and Parasitology, University of Ilorin, Ilorin, Nigeria; 4 Department of Microbiology, Obafemi Awolowo University, Ile-Ife, Nigeria; 5 Institute for Hygiene, University of Münster, Münster, Germany

**Keywords:** CC1-ST772-MRSA, CC8-ST789-MRSA, MRSA, Panton-Valentine Leukocidin, ST152-MRSA, *Staphylococcus aureus*

## Abstract

**Introduction:**

*Staphylococcus aureus* is a human pathogen associated with both hospital- and community-acquired infections. Data on its molecular epidemiology in North Central Nigeria are currently unavailable. This study investigated the antibiotic resistance, virulence genes, and the population structure of *S. aureus* in a tertiary hospital in North Central Nigeria.

**Methods:**

*S. aureus* strains from various clinical specimens collected between July 2017 and July 2021 were characterized based on antibiotic susceptibility testing and PCR detection of *nuc*, *mecA*, PVL (*lukS*-PV/*lukF*-PV), and immune evasion (*scn*, *sak*, *chp*) genes. Genotyping of the strains was performed by *spa* typing and whole-genome sequencing (WGS) of representative strains.

**Results:**

Of the 169 *S. aureus* strains, 52% (*n* = 88) were recovered from wound infections. The resistance rates were highest for penicillin (*n* = 162; 96%), followed by trimethoprim-sulfamethoxazole (*n* = 128; 76%), tetracycline (*n* = 66; 39%), gentamicin (*n* = 54; 32%), ciprofloxacin (*n* = 53; 31%), erythromycin (*n* = 31; 18%), and clindamycin (*n* = 3; 2%). Overall, 90 (53%) were *mecA*-positive (MRSA), and 81 strains (48%) were multidrug-resistant (MDR). Genotyping revealed a diverse *S. aureus* population, comprising 37 *spa* types and 19 sequence types (STs) in nine clonal complexes (CC1, CC5, CC8, CC15, CC22, CC30, CC45, CC88, CC121) and two singletons (ST152 and ST7572). The predominant MRSA lineages comprised CC1-ST772-MRSA-V-t657, CC8-ST789-MRSA-V-t091, and ST152-MRSA-Vc-t4690, while the common methicillin-susceptible *S. aureus* (MSSA) lineages were ST152-MSSA-t355, CC15-ST15-MSSA-t084, and CC5-ST5-MSSA-t311. The *dfrG* gene was detected across lineages, and 65% of all strains (MSSA: 68%; MRSA: 62%) were PVL-positive. Phylogenetic analysis revealed strain diversity, suggesting the dissemination of different lineages across various wards within the hospital.

**Conclusion:**

The *S. aureus* population at a tertiary hospital in North-Central Nigeria is highly diverse. The detection of PVL-positive ST152-MRSA-Vc and MDR CC1-ST722-MRSA-V is of significant public health concern. Genomic surveillance of *S. aureus*, combined with clinical data, is essential for effective infection prevention and control strategies.

## 1 Introduction

*Staphylococcus aureus* is a major human pathogen responsible for various infections, ranging from mild skin and soft tissue infections (SSTIs) to severe illnesses, e.g., sepsis, pneumonia, osteomyelitis, and food poisoning (Tong et al., 2015). These infections occur in hospitals and communities, leading to significant health and economic burdens (Noskin et al., 2007). *S. aureus* acquires antibiotic resistance through chromosomal mutations or via mobile genetic elements (Mlynarczyk-Bonikowska et al., 2022) and its pathogenicity is further enhanced by numerous virulence factors (Cheung et al., 2021). *S. aureus* infections represent a global public health concern, with a high burden in sub-Saharan Africa (Naghavi et al., 2024). A major factor contributing to this burden is the high prevalence (>70%) of Panton-Valentine Leukocidin (PVL)-positive *S. aureus*, particularly methicillin-susceptible *S. aureus* (MSSA), in Africa (Schaumburg et al., 2014). In contrast, 25% of *S. aureus* strains in Europe are PVL-positive (Bouchiat et al., 2017). PVL-positive *S. aureus* strains are associated with severe SSTIs, necrotizing pneumonia, life-threatening infections in children, and prolonged hospital stays (Garbo et al., 2024; Da Qu et al., 2022).

Methicillin-resistant *Staphylococcus aureus* (MRSA) is a key antimicrobial-resistant pathogen identified by the World Health Organization as a major antimicrobial resistance threat, particularly in relation to the Sustainable Development Goals (SDGs) on health (SDG 3) (Gajdács et al., 2021). MRSA is characterized by resistance to β-lactam agents, except the fifth-generation cephalosporins (ceftaroline and ceftobiprole), and often possesses additional resistance genes to other classes of antibiotics (Lakhundi and Zhang, 2018). The prevalence of MRSA varies geographically, ranging from 24 to 50% across Africa (Falagas et al., 2013; Lakhundi and Zhang, 2018). In Nigeria, MRSA rates vary by region: 42% in the South–South (Obasuyi et al., 2020), <60% in the South-West (Adeyanju et al., 2022; Olalekan et al., 2025) and 31% in the North-East (Bilyaminu et al., 2025).

The population structure of *S. aureus* is influenced by recombination, genetic mutation, and horizontal gene transfer. These phenomena result in diverse clones with varying antibiotic resistance and virulence profiles, capable of adapting and spreading in hospital and community settings (Souza et al., 2024). Studies indicate that the *S. aureus* population varies between and within regions and can change over time (Chen et al., 2021; Wang et al., 2022). The predominant African MRSA lineages include clonal complexes (CC)1, CC5, CC8, CC30, and CC88 (Lawal et al., 2022). In Nigeria, the dominant *S. aureus* lineages include CC5-MRSA, CC8-MRSA, CC15-MSSA, CC88-MRSA, CC121-MSSA, and ST152-MSSA (Shittu et al., 2011; Obasuyi et al., 2020; Adeyanju et al., 2022; Lawal et al., 2022).

Despite several regional studies, data on the molecular epidemiology of *S. aureus* in North Central Nigeria remain scarce. Therefore, the objectives of this study were to investigate the antibiotic resistance and virulence gene determinants, as well as the molecular epidemiology of *S. aureus* strains at the University of Ilorin Teaching Hospital (UITH).

## 2 Materials and methods

This study was conducted at UITH, a 640-bed tertiary referral centre in North Central Nigeria, with an average of ~12,000 inpatients and 126,000 outpatients annually. The inclusion criterion was non-duplicate *S. aureus* strains from diverse clinical specimens submitted to the Medical Microbiology Laboratory between July 2017 and July 2021. *S. aureus* strains from samples without a clinical diagnosis or from colonized patients in surveillance investigations were excluded. Relevant socio-demographic and clinical data were retrieved from laboratory records.

### 2.1 Identification and antibiotic susceptibility testing of the *Staphylococcus aureus* strains

Strains were presumptively identified using standard microbiological methods, including Gram staining, catalase, coagulase, DNase, mannitol fermentation, and lysostaphin susceptibility tests (Mast Group, UK). *S. aureus* strains were confirmed by MALDI-TOF MS (Sirius One, Bruker Daltonics, Bremen, Germany). Antimicrobial susceptibility testing was performed using the VITEK 2 automated system (bioMérieux, France), and clinical breakpoints were interpreted according to EUCAST (v12) clinical guidelines. Multidrug resistance (MDR) was defined as resistance to ≥3 antibiotic classes (Magiorakos et al., 2012).

### 2.2 PCR detection of *nuc*, *mecA*, non-ribosomal peptide synthetase (NRPS), *lukS-*PV/*lukF*-PV, *chp*, *sak*, and *scn* genes

Genomic DNA was extracted using the QIAamp tissue kit (QIAGEN, GmbH, Hilden, Germany), with lysostaphin added during the lysis step. Species identification as *S. aureus* and the methicillin resistance phenotype, i.e., MRSA, were determined by PCR detection of *nuc* and *mecA*, respectively (Brakstad et al., 1992; Murakami et al., 1991). PCR detection of the non-ribosomal peptide synthetase (NRPS) gene was performed to identify members of the *S. aureus* complex (Zhang et al., 2016). All strains were screened for the PVL toxin (*lukS-PV/lukF-PV*) gene (Lina et al., 1999) and immune evasion cluster (*scn*, *sak*, *chp*) genes (Van Wamel et al., 2006).

### 2.3 Molecular typing of *Staphylococcus aureus* strains

*Spa* typing (Harmsen et al., 2003) was done as previously described (Mellmann et al., 2006), and data were analyzed using Ridom SeqSphere software (version 7, Ridom GmbH, Münster, Germany). Representative strains (one strain per *spa* type) were selected for whole-genome sequencing (WGS). After DNA isolation (NEB Monarch Genomic Purification Kit, New England Biolabs, Ipswich, MA, USA), WGS was performed on a PacBio® Sequel IIe system (Pacific Biosciences, Menlo Park, CA, USA) using the SMRTbell® Express Template Prep Kit 2.0 (Pacific Biosciences Inc.). The sequencing data were assembled *de novo* using the SMRT® Link software suite v10 with default parameters and annotated using Prokka (1.14.6) on the Galaxy platform (Seemann, 2014). Using default parameters, antimicrobial resistance and virulence genes, with sequence types (STs), were predicted *in silico* with SeqSphere+ (v9).

### 2.4 SCC*mec* typing and phylogenetic analysis

The staphylococcal cassette chromosome *mec* (SCC*mec*) types (and subtypes) of the strains were determined using the Centre for Genomic Epidemiology tools, SCC*mec*Finder 1.2^1^. A pan-genome was constructed using Roary (v. 3.13.0) (Page et al., 2015) to identify core genes shared across all strains. Core gene alignment (present in 99% of strains) was generated using default parameters, including a minimum percentage identity of 95% for clustering homologous genes. Core gene sequences were also aligned using MAFFT (7.526) with the auto-selected strategy (Katoh and Standley, 2013). A maximum-likelihood phylogeny was inferred from the untrimmed alignment using IQ-Tree (v. 2.4.0) (Nguyen et al., 2014) under the general time-reversible substitution model with 1,000 ultrafast bootstrap replicates for branch and node support. The visualization and annotation of the phylogenetic tree were performed using iTOL (v6.7.4) (Letunic and Bork, 2021). All analyses were conducted on the public Galaxy server^2^. Genome sequences of the representative strains were deposited on the NCBI website under Bioproject accession number PRJNA1463733.

### 2.5 Data analysis

The Chi-Square test and Student’s t-test were used to compare antibiotic resistance rates and PVL prevalence between MRSA and MSSA. Strains with the category ‘susceptible, increased exposure’ were grouped as ‘susceptible’ when comparing resistance rates between groups. A *p*-value <0.05 was considered statistically significant.

## 3 Results

### 3.1 Distribution of *Staphylococcus aureus* associated with clinical infections

A total of 169 *S. aureus* strains were obtained from inpatients and outpatients (Table 1). The cohort comprised 87 male and 82 female patients, with mean ages of 24 ± 21.9 and 29.4 ± 20.9 years, respectively. Strains were predominantly recovered from wound infections (*n* = 88; 52%) and bloodstream infections (*n* = 32; 19%), followed by upper respiratory tract and urogenital infections (*n* = 13; 8% each), eye infections (*n* = 11; 7%), lower respiratory tract infections (*n* = 7; 4%), and septic arthritis (*n* = 5; 3%).

**Table 1:** Demographic and clinical characteristics of *S. aureus* strains from UITH, Nigeria.

Characteristics	*S. aureus* (*n* = 169)	
	MRSA: *n* = 90 (%)	MSSA: *n* = 79 (%)
Age stratification		
≤ 14	26 (29)	37 (47)
15–28	13 (14)	12 (15)
29–43	27 (30)	12 (15)
44–58	17 (19)	10 (13)
> 58	7 (8)	8 (10)
Sex		
Male	42 (47)	45 (57)
Female	48 (53)	34 (43)
Location		
In-patient	62 (69)	55 (70)
Out-patient	28 (31)	24 (30)
Ward/Clinic		
Accident & Emergency	11 (12)	11 (14)
Ear, Nose, and Throat	3 (3)	6 (8)
Emergency pediatric	7 (8)	17 (22)
Female medical	8 (9)	3 (4)
Female surgical	9 (10)	2 (2)
General outpatient	15 (17)	11 (14)
Male Medical	3 (3)	4 (5)
Male surgical	6 (7)	3 (4)
Neonatal intensive care	8 (9)	11 (14)
National health insurance scheme	1 (1)	3 (4)
Obstetrics & gynecology	8 (9)	2 (2)
Orthopedics	2 (2)	0 (0)
Pediatric surgical	5 (6)	2 (2)
Surgical outpatient	4 (4)	3 (4)
Sickle cell	0 (0)	1 (1)
Clinical diagnosis		
Blood stream infections^a^	12 (13)	20 (25)
Eye infections^b^	5 (6)	6 (8)
LRTIs^c^	4 (4)	3 (4)
URTIs^d^	6 (7)	7 (9)
Septic arthritis	2 (2)	3 (4)
Urogenital infections^e^	9 (10)	4 (5)
Wound infections^f^	52 (58)	36 (45)
Specimen		
Aspirate	2 (2)	3 (4)
Blood	12 (13)	20 (25)
Eye discharge	5 (6)	6 (8)
LRTI sample^g^	4 (4)	3 (4)
URTI samples^h^	6 (7)	7 (9)
Urogenital sample^i^	9 (10)	4 (5)
Wound sample^j^	52 (58)	36 (45)

a Endocarditis, septicaemia, neonatal sepsis, meningitis.

b Ophthalmia neonatorum, conjunctivitis.

c Pleuritis, parapneumonic infection, community-acquired pneumonia, hospital-acquired pneumonia.

d Upper respiratory tract infections (otitis media, rhinosinusitis, sinusitis, and pharyngotonsillitis).

e Urethritis, puerperal sepsis, vaginitis, pelvic inflammatory disease, and urinary tract infections.

f Abscess, catheter line infection, wound ulcer, burn infection, surgical site infection, mastitis, pyomyositis, osteomyelitis, diabetes mellitus foot syndrome, implant infections, skin and soft tissue infections (necrotizing fasciitis, cellulitis, folliculitis, abscess, paronychia).

g Sputum, pleural fluid.

h Ear discharge, nasal discharge, and pharngotonsillar swab. ^i^Urine, urethral swab, high vaginal swab, endocervical swab.

j Wound biopsy, pus, exudates, deep wound swab, wound discharge.

### 3.2 Antibiotic susceptibility of *Staphylococcus aureus* strains

All the strains were susceptible to daptomycin, fosfomycin, fusidic acid, linezolid, mupirocin, rifampicin, glycopeptides, and tigecycline. High resistance rates were recorded for penicillin (*n* = 162; 96%) and trimethoprim-sulfamethoxazole (*n* = 128; 76%, Table 2). Overall, 53% (*n* = 90) of strains were *mecA*-positive, including one phenotypically susceptible strain to cefoxitin. MRSA strains demonstrated significantly higher rates than MSSA strains for gentamicin (59% vs. 1%), fluoroquinolones (53% vs. 6%), and tetracycline (50% vs. 27%). Multidrug resistance was observed in 48% of strains (*n* = 81), predominantly among MRSA (74% vs. 18% in MSSA). The inducible clindamycin resistance rate was low (2%).

**Table 2:** Antibiotic susceptibility testing of *S. aureus* strains from clinical samples at UITH, Nigeria.

Antibiotics	MRSA (*n* = 90)		MSSA (*n* = 79)		*p*-value
	Resistance (%)	Susceptible (%)	Resistance (%)	Susceptible (%)	
Cefoxitin	89 (99)	1 (1)	0 (0)	79 (100)	**-**
Penicillin	90 (100)	0 (0)	72 (91)	7 (9)	**0.001**
Co-amoxiclav	89 (99)	1 (1)	4 (5)	75 (95)	**0.001**
Gentamicin	53 (59)	37 (41)	1 (1)	78 (99)	**0.001**
Levofloxacin	48 (53)	42 (47)	5 (6)	74 (94)	**0.001**
Ciprofloxacin	48 (53)	42 (47)	5 (6)	74 (94)	**0.001**
Moxifloxacin	48 (53)	42 (47)	5 (6)	74 (94)	**0.001**
Azithromycin	28 (31)	62 (69)	3 (4)	76 (96)	**0.001**
Clarithromycin	28 (31)	62 (69)	3 (4)	76 (96)	**0.001**
Erythromycin	28 (31)	62 (69)	3 (4)	76 (96)	**0.001**
Clindamycin	3 (3)	87 (97)	0 (0)	79 (100)	-
Tetracycline	45 (50)	45 (50)	21 (27)	58 (73)	**0.001**
Trimethoprim-Sulfamethoxazole	74 (82)	16 (18)	54 (68)	25 (32)	**0.026**
Linezolid	0 (0)	90 (100)	0 (0)	79 (100)	-
Daptomycin	0 (0)	90 (100)	0 (0)	79 (100)	-
Teicoplanin	0 (0)	90 (100)	0 (0)	79 (100)	-
Vancomycin	0 (0)	90 (100)	0 (0)	79 (100)	-
Tigecycline	0 (0)	90 (100)	0 (0)	79 (100)	-
Fosfomycin	0 (0)	90 (100)	0 (0)	79 (100)	-
Fusidic acid	0 (0)	90 (100)	0 (0)	79 (100)	-
Mupirocin	0 (0)	90 (100)	0 (0)	79 (100)	-
Rifampicin	0 (0)	90 (100)	0 (0)	79 (100)	-

Bold values represent statistically significant p-values for resistance between MRSA and MSSA to the corresponding antibiotics.

### 3.3 PCR detection of the immune evasion cluster and the bi-component (lukF-PV/lukS-PV) genes

The prevalence of immune evasion cluster genes (*scn*, *sak,* and *chp*) was 94, 66, and 25%, respectively. The PVL gene was present in 110 strains (65%), with MSSA and MRSA at 68 and 62%, respectively (Supplementary Table 1), with no statistically significant difference (*p* = 0.4).

### 3.4 Molecular typing of *Staphylococcus aureus* strains by spa typing

The *spa* typing identified 37 genotypes, including one new *spa* type (t20210). The most common were t355 (23%), t091, and t657 (11% each, Table 3). Among the MRSA strains, the dominant types were t657 (20%), t4690 (18%), and t091 (17%). In MSSA, t355 was most common (44%), followed by t084 (13%) and t311 (8%) (Table 3).

**Table 3:** Characterization of MRSA and MSSA strains from UITH, Nigeria, based on *spa* typing and MLST.

Clonal complex (*n*)	Sequence types from WGS data (*n*)	*spa* types (*n*)	MRSA *n* (%)	MSSA *n* (%)
CC1 (28)	ST1 (7)	**t114** (1), **t1931** (2), t1931 (1), **t5557** (2), t20210* (1)	6 (86)	1 (14)
	ST772 (21)	**t657** (18), t657 (1), **t9180** (1), **t13180** (1)	20 (95)	1 (5)
CC5 (9)	ST5 (8)	**t311** (1), t311 (6), t450 (1)	1 (14)	7 (86)
	ST6 (1)	t304 (1)	0 (0)	1 (100)
CC8 (32)	ST8 (12)	**t008** (4), t008 (1), **t064** (3), **t1476** (3), **t11148** (1)	11 (92)	1 (8)
	ST789 (20)	**t091** (15), t091 (4), **t2616** (1)	16 (80)	4 (20)
CC15 (13)	ST15 (11)	t084 (10), t5126 (1)	0 (0)	11 (100)
	ST3712 (2)	t326 (2)	0 (0)	2 (100)
CC22 (1)	ST22 (1)	**t2933** (1)	1 (100)	0 (0)
CC30 (5)	ST30 (3)	t13518 (3)	0 (0)	3 (100)
	ST243 (2)	t021 (2)	0 (0)	2 (100)
CC45 (2)	ST508 (1)	t1156 (1)	0 (0)	1 (100)
	ST2498 (1)	t861 (1)	0 (0)	1 (100)
CC88 (7)	ST88 (7)	**t2393** (1), **t1244** (2), **t7558** (2), **t13249** (2)	7 (100)	0 (0)
CC121 (13)	ST121 (11)	**t314** (5), t314 (5), **t1991** (1)	6 (55)	5 (45)
	ST7570** (1)	t159 (1)	0 (0)	1 (100)
	ST7571** (1)	t159 (1)	0 (0)	1 (100)
	ST152 (58)	t355 (34), **t355** (4), **t1172** (1), t1299 (1), t4687 (1), **t4690** (16), **t11375** (1)	22 (38)	36 (62)
	ST7572** (1)	t355 (1)	0 (0)	1 (100)

MRSA identified with *spa* types in bold; *, new *spa* type; **, new sequence types.

### 3.5 Identification of *Staphylococcus aureus* clonal lineages based on MLST

WGS was performed on randomly chosen strains representing the 37 *spa* types. These *S. aureus* strains comprised 23 MRSA and 14 MSSA. We identified 19 STs, including three new types: ST7570, ST7571, and ST7572 (Table 3). The most frequently occurring STs were ST152 (34%), ST772 (12%), ST789 (12%), ST8, ST15, and ST121 (7% each), and ST5 (5%). ST1, ST30, ST88, ST243 and ST3712 occurred at lower frequencies (≤4%), and ST6, ST22, ST508, ST2498, ST7570, ST7571, ST7572 were represented by a single strain (<1%, Table 3). The strains were grouped into nine CCs, with CC8 (*n* = 32), CC1 (*n* = 28), CC15, and CC121 (*n* = 13 each) being the most common. Others include CC5 (*n* = 9), CC88 (*n* = 7), CC30 (*n* = 5), CC45 (*n* = 2), CC22 (*n* = 1), and two singletons: ST152 (*n* = 58) and ST7572 (*n* = 1). Diverse *S. aureus* lineages (>4 CCs) were observed in some clinical presentations, particularly bloodstream, upper respiratory tract, urogenital, and wound infections. *S. aureus* strains assigned to CC1 and ST152 were identified in all categories of clinical presentations, whereas those grouped with CC8 were observed in all infection groups except eye infections (Table 4).

**Table 4:** Prevalence of *S. aureus* clonal lineages according to clinical diagnosis.

Clinical diagnosis	CC	ST	Frequency (%)
Bloodstream infection^a^ (*n* = 32)	CC1	**ST1** (3), **ST772** (1)	4 (13)
	CC5	ST5 (1)	1 (3)
	CC8	**ST8** (1), **ST789** (4)	5 (16)
	CC15	ST15 (3), ST3712 (1)	4 (13)
	CC30	ST30 (2), ST243 (2)	4 (13)
	CC88	**ST88** (2)	2 (6)
	CC121	**ST121** (3)	3 (9)
		**ST152** (9)	9 (28)
Eye infection^b^ (*n* = 11)	CC1	**ST1** (2), ST772 (1)	3 (27)
	CC121	**ST121** (2)	2 (18)
		**ST152** (6)	6 (55)
LRTIs^c^ (*n* = 7)	CC1	**ST772** (2)	2 (29)
	CC8	**ST8** (2)	2 (29)
	CC15	ST15 (1)	1 (14)
		ST152 (2)	2 (29)
URTIs^d^ (*n* = 13)	CC1	**ST1** (1)	1 (8)
	CC5	**ST5** (1), ST6 (1)	2 (15)
	CC8	**ST8** (1)	1 (8)
	CC15	ST3712 (1)	1 (8)
	CC88	**ST88** (1)	1 (8)
	CC121	ST121 (1)	1 (8)
		**ST152** (5) ST7572 (1)	5 (38) 1 (8)
Septic arthritis (*n* = 5)	CC1	**ST772** (1)	1 (20)
	CC8	ST789 (1)	1 (20)
	CC121	ST7571 (1)	1 (20)
		ST152 (2)	2 (40)
Urogenital infection^e^ (*n* = 13)	CC1	ST1 (1), **ST772** (3)	4 (31)
	CC5	ST5 (1)	1 (8)
	CC8	**ST789** (2)	2 (15)
	CC15	ST15 (1)	1 (8)
	CC22	ST22 (1)	1 (8)
	CC45	ST45 (1)	1 (8)
		**ST152** (3)	3 (23)
Wound infection^f^ (*n* = 88)	CC1	**ST772** (13)	13 (15)
	CC5	ST5 (5)	5 (6)
	CC8	**ST8** (9), **ST789** (13)	22 (25)
	CC15	ST15 (6)	6 (7)
	CC30	ST30 (1)	1 (1)
	CC45	ST508 (1)	1 (1)
	CC88	**ST88** (4)	4 (5)
	CC121	**ST121** (5), ST7570 (1)	6 (7)
		**ST152** (30)	30 (34)

Sequence Types in which MRSA strains were identified are in bold format.

a Endocarditis, septicaemia, neonatal sepsis, meningitis.

b Ophthalmia neonatorum, conjunctivitis.

c Pleuritis, parapneumonic infection, community-acquired pneumonia, hospital-acquired pneumonia.

d Upper respiratory tract infections (otitis media, rhinosinusitis, sinusitis, and pharyngotonsillitis).

e Urethritis, puerperal sepsis, vaginitis, pelvic inflammatory disease, and urinary tract infections.

f Abscess, catheter line infection, wound ulcer, burn infection, surgical site infection, mastitis, pyomyositis, osteomyelitis, diabetes mellitus foot syndrome, implant infections, skin and soft tissue infections (necrotizing fasciitis, cellulitis, folliculitis, abscess, paronychia).

MRSA was predominant in CC1, CC8, and CC88, whereas MSSA were mainly associated with CC15, CC30, and CC45 (Table 3). Both MRSA and MSSA were mainly identified in CC121 and ST152. Of the 23 MRSA strains selected for WGS, the following SCC*mec* types were identified: Vc (48%), V (22%), IVa (26%), and XIII (4%). SCC*mec* elements were distributed across the CCs, notably IV/IVa in CC8, CC22, CC88, and CC121; V in CC1-ST772 and CC8-ST789; and Vc in CC1-ST1, CC8, CC88, and ST152. Moreover, one ST152 strain possessed the SCC*mec* XIII element (Table 5).

**Table 5:** Characterization of the *S. aureus* strains from UITH, Nigeria, based on clonal lineages, SCC*mec* typing, and detection of antibiotic resistance and virulence genes.

Strain ID	CC	ST	SCC*mec*	*spa*	GEN	ERY	QUN	TET	SXT	LEU	EXF	TST	ENT	HLB	ADH
SW34018	1	1	Vc	t114	-	-	-	*tet(K)*	-	*lukD, lukS-PV/lukF-PV*	-	-	*seh*	-	*fnbA, cna, clfA, clfB*
BC05119	1	1	Vc	t1931	-	-	-	*tet(K)*	-	*lukD, lukS-PV/lukF-PV*	-	-	*seh*	-	*fnbA, cna, clfA*
SW495	1	1	Vc	t557	-	-	-	*tet(K)*	-	*lukD, lukS-PV/lukF-PV*	-	-	*seh*	-	*fnbA, cna, clfA, clfB*
BC61520	1	1	Vc	t20210	-	-	-	*tet(K)*	-	*lukD, lukS-PV/lukF-PV*	-	-	*seh*	-	*fnbA, cna, clfB*
SW975	1	772	V	t657	*aac(6′)-Ie/aph(2″)-Ia*	*mph(C)/msr(A)*	*gyrA_S84L/parC_S80F*	-	*dfrG*	*lukS-PV/lukF-PV*	-	-	*sea, sec*	-	*clfA*
SW04121	1	772	V	t9180	*aac(6′)-Ie/aph(2″)-Ia*	*mph(C)/msr(A)*	*gyrA_S84L/parC_S80F*	-	*dfrG*	*lukS-PV/lukF-PV*	-	-	*sea, sec*	-	*clfA*
SP37518	1	772	V	t1318	*aac(6′)-Ie/aph(2″)-Ia*	*mph(C)/msr(A)*	*gyrA_S84L/parC_S80F*	-	*dfrG*	*lukS-PV/lukF-PV*	-	-	*sea, sec*	-	*clfA*
SW69220	5	5	-	t311	-	-	-	-	-	*lukD, lukS-PV/lukF-PV*	-	-	-	-	-
SW04521	5	5	-	t450	-	-	-	-	*dfrG*	*lukD*	-	-	*sec*	-	*fnbB*
SW115017	5	6	-	t304	-	-	-	*tet(M)*	-	*lukD*	-	-	*sea*	-	-
SW14118	8	8	Vc	t008	*aac(6′)-Ie/aph(2″)-Ia*	-	*gyrA_S84L/parC_S80F*	*tet(K)*	*dfrG*	*lukD*	-	-	*seb*	*hlb*	*fnbA*
SP11218	8	8	Vc	t064	-	-	*gyrA_S84L/parC_S80F*	*tet(K)*	*dfrG*	*lukD*	-	-	*sea, seb*	-	*fnbA*
SWI5020	8	8	IV	t1476	*aac(6′)-Ie/aph(2″)-Ia*	*erm(C)*	*gyrA_S84L/parC_S80F*	*tet(K)*	*dfrC/dfrG*	*lukD*	-	*tst-1*	-	-	*fnbA*
SW10021	8	8	IVa	t11148	*aac(6′)-Ie/aph(2″)-Ia*	-	*gyrA_S84L/parC_S80F*	*tet(K)*	*dfrC/dfrG*	*lukD*	-	*tst-1*	-	-	*fnbA*
BC96119	8	789	V	t091	*aac(6′)-Ie/aph(2″)-Ia*	-	*gyrA_S84L/parC_S80F*	-	*dfrG*	*lukD*	-	-	-	*hlb*	*clfB*
SW934	8	789	V	t2616	*aac(6′)-Ie/aph(2″)-Ia*	-	*gyrA_S84L/parC_S80F*	*tet(K)*	*dfrG*	*lukD*	-	-	-	*hlb*	*clfB*
BC83318	15	15	-	t084	-	-	-	-	*dfrG*	*lukD*	-	-	-	-	*clfA, clfB*
SW10121	15	15	-	t5126	-	-	-	-	*dfrG*	*lukD*	*eta*	-	-	-	-
SW973	15	3712	-	t326	-	-	-	-	*dfrG*	*lukD*	*eta, etb*	-	-	-	*clfB*
SW286	22	22	IVa	t2933	-	-	-	-	*dfrC*	-	-	*tst-1*	*sea*	-	*fnbA, cna*
SW50319	30	30	-	t13518	-	-	-	-	*dfrG*	*lukS-PV/lukF-PV*	-	-	-	*-*	*cna*
BC50519	30	243	-	t021	-	-	-	-	-	*lukS-PV/lukF-PV*	-	-	-	*hlb*	*cna*
SW296	45	508	-	t1156	-	-	-	-	-	-	-	*tst-1*	*sec*	-	*cna*
UR1844	45	2498	-	t861	-	-	-	-	-	-	-	*tst-1*	*seb, sec*	-	*clfB*
BC08919	88	88	Vc	t2393	-	-	-	*tet(K)*	-	*lukD, lukS-PV/lukF-PV*	-	-	*sec*	-	-
SW72920	88	88	IVa	t7558	-	-	-	-	*dfrC*	*lukD*	-	-	-	-	*clfA*
BC04219	88	88	Vc	t12444	-	-	-	-	*dfrG*	*lukD, lukS-PV/lukF-PV*	-	-	*sec*	-	-
SW17819	88	88	IVa	t13249	-	-	-	-	*dfrC*	*lukD*	-	-	-	-	*clfA*
SW10221	121	121	IVa	t1991	-	-	-	*tet(K)*	*dfrG*	*lukD, lukS-PV/lukF-PV*	-	-	*seb*	-	*cna*
SW78320	121	121	-	t314	-	-	-	-	-	*lukD, lukS-PV/lukF-PV*	-	-	*seb*	-	*cna*
SW53320	121	7571	-	t159	-	-	-	-	*dfrG*	*lukD, lukS-PV/lukF-PV*	-	-	*seb*	-	*cna*
SW08821	-	152	XIII	t355	*aac(6′)-Ie/aph(2″)-Ia*	-	-	-	*-*	*lukD, lukS-PV/lukF-PV*	-	-	-	*hlb*	-
SW923	-	152	-	t1299	-	-	-	-	*dfrG*	*lukD, lukS-PV/lukF-PV*	-	-	-	*hlb*	*fnbB, cna*
SW14218	-	152	-	t4687	-	-	-	-	*dfrG*	*lukD, lukS-PV/lukF-PV*	-	-	-	*hlb*	*fnbB*, *cna*
BC62020	-	152	Vc	t4690	-	-	-	*tet(K)*	*dfrG*	*lukD, lukS-PV/lukF-PV*	-	-	-	*hlb*	-
SW857	-	152	Vc	t1172	-	-	-	*tet(K)*	*dfrG*	*lukD, lukS-PV/lukF-PV*	-	-	-	*hlb*	*fnbB, cna*
SW93917	-	152	Vc	t11375	-	-	-	*tet(K)*	*dfrG*	*lukD, lukS-PV/lukF-PV*	-	-	-	*hlb*	-

ST, Sequence Type; CC, Clonal Complex; SCC*mec*, Staphylococcal Cassette Chromosome mec; GEN, Gentamicin; ERY, Erythromycin, QUN, Quinolones (Ciprofloxacin; Levofloxacin; Moxifloxacin); TET, Tetracycline; SXT, Trimethoprim-Sulfamethoxazole; LEU, Leukocidin genes (*lukD*, leukocidin D; *lukF/S-PV*, Panton-Valentine leukocidin subunit F; *lukS-PV*, Panton-Valentine leukocidin subunit S); EXF, Exfoliative toxin genes (*eta*, exfoliative toxin A; *etb*, exfoliative toxin B); TST, Toxic shock syndrome toxin (*tst*-1); ENT, Enterotoxins (*sea*, enterotoxin A; *seb*, enterotoxin B; *sec*, enterotoxin C; *seh*, enterotoxin H); HLB, β-haemolysin; ADH, Adhesion genes (*clfA*, clumping factor A; *clfB*, clumping factor B; *cna*, collagen adhesin; *fnbA*, fibronectin-binding protein A; *fnbB*, fibronectin-binding protein B); antibiotic resistance genes, [variants of SCC*mec*, IVa, Vc, and XIII; resistance to aminoglycosides, aac(6′)-Ie/aph(2″)-Ia; a bi-functional aminoglycoside acetyltransferase and phosphotransferase;* mph(C)/msr(A)*, macrolide phosphotransferase/efflux pump; *erm(C)*, macrolide, lincosamide, and type B streptogramin ribosomal site modification; *lnu(A)*, lincosamide nucleotidyltransferase; *gyrA_S84L/parC_S80F*, fluoroquinolone point-mutated DNA gyrase and topoisomerase IV; *tet(38)/tet(K)*, tetracycline efflux pump; *dfrC/drfG*, dihydrofolate reductase enzyme].

### 3.6 Characterization of the *Staphylococcus aureus* clonal lineages based on some antibiotic resistance and virulence genes

Some STs possessed diverse AMR and virulence genes, and some genes were associated with specific STs (Table 5). Within CC1, SCC*mec* typing differentiated ST1 (SCC*mec*Vc) from ST772 (SCC*mec*V). The ST772 strains possessed resistance genes for erythromycin (*mph(C)/msr(A)*), gentamicin (*aac(6′)-Ie-aph(2″)-Ia*), trimethoprim (*dfrG*), and fluoroquinolones (*gyrA* S84L, *parC* S80F), with the enterotoxins (*sea*, *sec*). These genes were absent in the ST1 lineage, which instead harbored *tetK* and some virulence genes (*cna*, *fnbA, seh*, *lukD*). CC8, comprising ST8 and ST789, harbored several antibiotic resistance genes and was *lukD*-positive; ST8 was *fnbA*-positive, whereas ST789 was *clfB*-positive. All CC30 strains were PVL- and *cna*-positive, while the CC45 strains were *tst*-positive. All the CC88 strains were MRSA. Moreover, CC88-MRSA-IVa strains harbored *dfrC*, *clfA*, and *lukD*, whereas CC88-MRSA*-*Vc strains were positive for PVL, *lukD*, and *sec* genes. All CC121 strains harbored PVL, *lukD*, *seb*, and *cna*, while ST152 strains were positive for PVL, *lukD*, and *hlb* (Table 5).

### 3.7 Genomic epidemiology of *Staphylococcus aureus* strains

Phylogenetic analysis of *S. aureus* revealed a genetically diverse population comprising various CCs and SCC*mec* elements (Figure 1). The ST152 lineage was the most heterogeneous, encompassing both MSSA (e.g., t1299, t4687) and MRSA (e.g., t355, t1172, t4690, t1375) associated with SCC*mec*Vc and XIII. Three major MRSA lineages were identified. The first, CC8 (ST8-MRSA), included strains with different *spa* types (t008, t064, t1476, t11148) and SCC*mec* IV and Vc. The second, CC88 (ST88), comprised strains with *spa* types t12444, t2393, t7558, and t13249, with SCC*mec*IVa and Vc. CC1-ST1-MRSA-Vc strains formed a distinct cluster linked to *spa* types t114, t5557, t20210, and t1931. Other clusters included CC1-ST772-MRSA-V and CC8-ST789-MRSA-V. The distribution of various CCs across clinical wards indicates a diverse *S. aureus* population within the hospital. Specifically, PVL-positive MRSA strains assigned to CC1, CC88, and ST152-MRSA were identified in the NICU.

**Figure 1:**
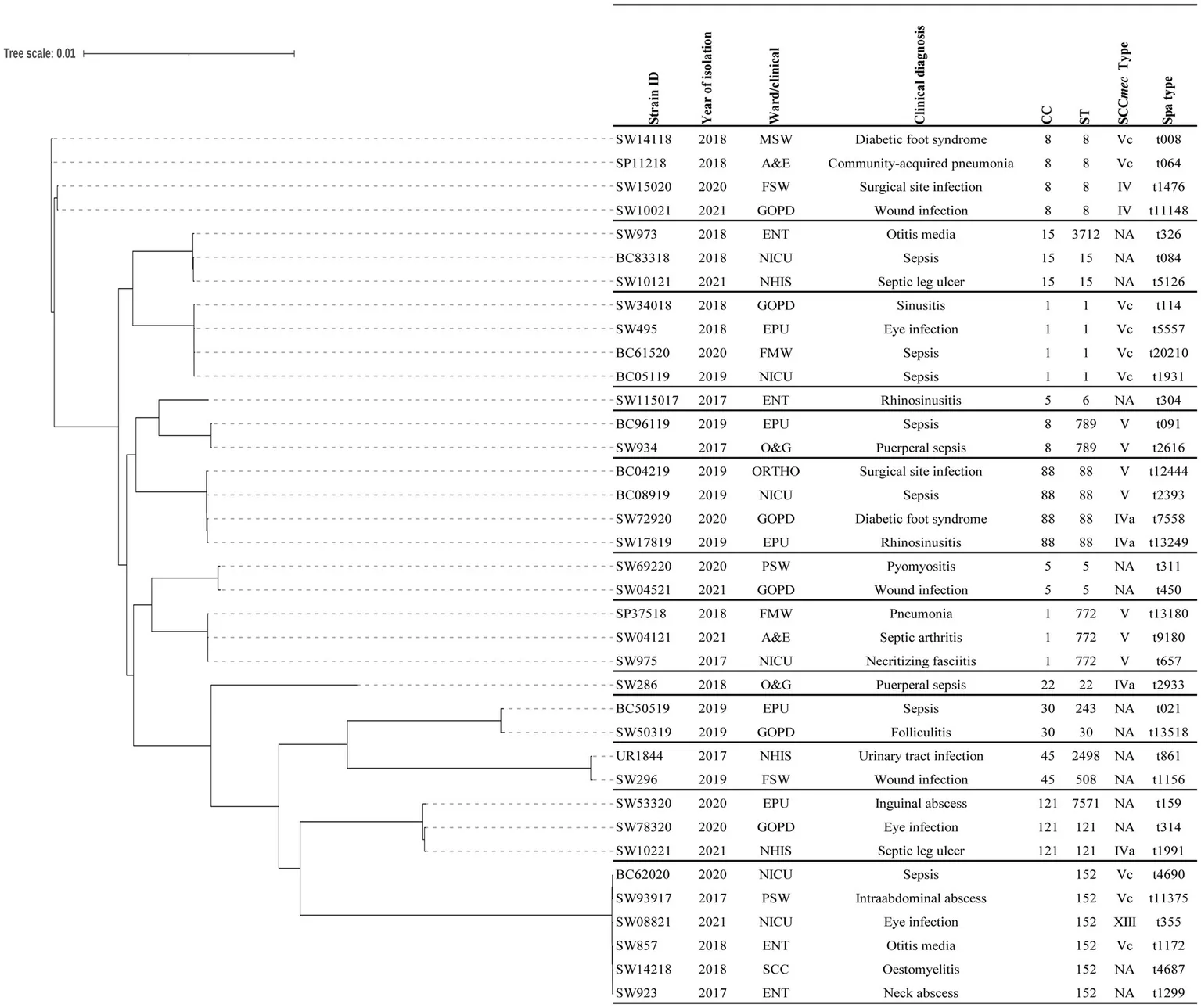
Maximum-likelihood phylogenetic tree among clinical *Staphylococcus aureus* strains recovered between 2017 and 2021. The tree was constructed from whole-genome sequence data and visualized using Interactive Tree of Life (iTOL). Strains are annotated with strain ID, year of isolation, hospital ward, clinical diagnosis, clonal complex (CC), sequence type (ST), *SCCmec* type, and *spa* type, NA: Not Applicable. A&E, Accident & Emergency; ENT, Ear, Nose, and Throat; EPU, Emergency Pediatric Unit; FSW, Female Surgical Ward; FMW, Female Medical Ward; GOPD, General Outpatient Department; MSW, Male Surgical Ward; NHIS, National Health Insurance Scheme; NICU, Neonatal Intensive Care Unit; O&G, Obstetrics & Gynecology; ORTHO, Orthopedic Ward; PSW, Pediatric Surgical Ward; PMW, Pediatric Medical Ward; SCC, Sickle Cell Clinic.

## 4 Discussion

Based on phenotypic and molecular methods, we investigated the antimicrobial resistance and virulence genes, as well as the population dynamics of *S. aureus* from clinical samples in North Central Nigeria. The most frequent clinical presentations were wound infections, and 58% of strains from this source were MRSA, which is similar to reports from other African settings (Monk et al., 2025; Wangai et al., 2019). All strains were susceptible to daptomycin, fosfomycin, fusidic acid, linezolid, mupirocin, rifampicin, glycopeptides, and tigecycline, corroborating previous Nigerian studies (Adeyanju et al., 2022; Shittu et al., 2011). Previous studies also reported that some of these antibiotics, such as fusidic acid, fosfomycin, linezolid, mupirocin, rifampicin, and tigecycline, are effective against *S. aureus* with low resistance rates (<3%) across various regions in Africa, including Ghana (Egyir et al., 2014), Nigeria (Adeyanju et al., 2022), Mozambique (Garrine et al., 2023), and Ethiopia (Verdú-Expósito et al., 2020). The low *S. aureus* resistance rates to these antibiotics may indicate their limited use due to high cost and lack of availability (Abubakar and Salman, 2024; Kallberg et al., 2018; Pauwels et al., 2021). Penicillins, cephalosporins, aminoglycosides, fluoroquinolones, sulfonamides, macrolides, and tetracyclines are the most common antibiotics prescribed in Nigeria (Abubakar and Salman, 2024; Egwuenu et al., 2024). This study documented a moderate *S. aureus* resistance rate (>30%) to tetracycline, gentamicin, the fluoroquinolones, and a high resistance rate (>90%) to penicillin and trimethoprim-sulfamethoxazole (>70%), which is comparable with regional data (Adeyanju et al., 2022; Garrine et al., 2023; Perovic et al., 2015; Shittu et al., 2011).

PVL-positive *S. aureus* strains are associated with SSTIs, necrotizing pneumonia, and severe sepsis, often leading to poor clinical outcomes, particularly in children (Garbo et al., 2024; Da Qu et al., 2022). PVL-positive *S. aureus* is endemic in Africa (Schaumburg et al., 2014). In this study, more than 60% of strains were PVL-positive, which is similar to reports from Southwest Nigeria (70%) (Olalekan et al., 2025) and The Gambia (61%) (Darboe et al., 2019). The prevalence of PVL-positive *S. aureus* varies across regions in Nigeria, including South–South (13–52%) (Ide et al., 2025; Obasuyi et al., 2020), South-West (13–82%) (Adeyanju et al., 2022; Ayepola et al., 2015), North Central (31%) (Essien et al., 2021), and North East Nigeria (10.9%) (Onanuga et al., 2021). Although PVL is typically more prevalent among MSSA in Africa (Olalekan et al., 2025; Schaumburg et al., 2014), this study found no significant difference (68% vs. 62%; *p* < 0.40). The high prevalence of PVL-positive *S. aureus* in North Central Nigeria reinforces its endemicity in Africa and underscores its clinical importance. Although PVL-positive *S. aureus* strains were detected in various clinical presentations (Supplementary File 1), the primary clinical diagnosis in this study was wound infections (including SSTIs), accounting for 52% of cases. PVL-positive *S. aureus* has been associated with SSTIs in Africa (Schaumburg et al., 2014) and may explain the high prevalence observed in this study. Host, pathogen, or environmental factors may contribute to the spread of PVL-positive *S. aureus* in Africa. A recent study indicated that the high prevalence of PVL-positive *S. aureus* in Africa could be attributed to the susceptibility of polymorphonuclear (PMN) cells in the African population to PVL and an increased inflammatory response (Grebe et al., 2025). This could lead to excessive PMN infiltration and lysis, enhanced tissue destruction, and necrotizing infections.

The dominant *S. aureus* lineages (CC1, CC8, and ST152) were identified in various clinical samples, which is similar to recent reports in Ghana and Nigeria (Egyir et al., 2024; Olalekan et al., 2025). CC1-ST772-MRSA (Bengal Bay clone) emerged in India, spreading locally in hospitals and communities before rapidly spreading worldwide through medical tourism (Steinig et al., 2019). In this study, MDR CC1-ST772-MRSA-V-t657 was the predominant MRSA lineage, consistent with recent reports on human nasal samples (Ogundipe et al., 2020) and clinical samples (Olalekan et al., 2025) in South-West Nigeria. It has been associated with necrotizing fasciitis (Sabat et al., 2022) and outbreaks at the NICU (Brennan et al., 2012) as well as SSTIs (Blomfeldt et al., 2017). Our study provides evidence that the prevalence of the MDR CC1-ST772-MRSA-V clone is increasing in Nigeria (O’Malley et al., 2015; Obasuyi et al., 2020; Ogundipe et al., 2020; Olalekan et al., 2025; Essien et al., 2021) after the first documented case over a decade ago (Raji et al., 2013). The MDR CC8-ST789-MRSA-V was also one of the common MRSA clones in this study. This lineage has been reported in Africa (Ogundipe et al., 2020; Olalekan et al., 2025; Schaumburg et al., 2022) including colonized individuals (Laurence Yehouenou et al., 2023) and patients with SSTIs (Schaumburg et al., 2022). It has also been linked with zoonotic transmission potential (Ogundipe et al., 2020). The emergence of CC8-ST789-MRSA-V in Nigeria, with its MDR phenotype and reported cases in both human and animal hosts, highlights the need for strengthened One Health surveillance to understand its transmission dynamics and public health significance.

The PVL-positive ST152 was the most common lineage (35%), including both MSSA and MRSA (Figure 1; Table 3). This clone has consistently been reported as among the dominant lineages in Africa, particularly in Central and West Africa (Lawal et al., 2022). The high proportion of MRSA, particularly ST152-MRSA-Vc-t4690, is noteworthy (Tables 3, 5). This observation is similar to recent findings in Nigeria (Olalekan et al., 2025) but differs from the predominant ST152-MRSA-Va-t355 identified in Ghana (Egyir et al., 2024) and Kenya (Nyasinga et al., 2024). Our study also identified an ST152-MRSA-XIII-t355 strain from a patient with an eye infection at the NICU, and likely the first report in North Central Nigeria. These observations suggest that ST152-MRSA may have emerged through multiple independent acquisitions of SCC*mec* elements rather than clonal expansion. Previous genomic studies have demonstrated that MRSA frequently emerged through the horizontal acquisition of SCC*mec* by MSSA progenitors, followed by clonal diversification under antimicrobial selection pressure (Deurenberg et al., 2007). The coexistence of MSSA and MRSA strains within the ST152 lineage supports the hypothesis of independent SCC*mec* acquisition events within an established MSSA background, a phenomenon previously documented in African *S. aureus* populations (Baig et al., 2020).

In this study, representative PVL-positive ST152 strains retained an intact haemolysin (*hlb*) gene (Table 5). While many human-adapted strains carry φSa3-like prophages that integrate into the *hlb* locus and introduce immune evasion cluster genes, not all PVL-positive *S. aureus* strains experience *hlb* disruption (Rohmer and Wolz, 2021). Some community-associated and livestock-adapted strains retain an intact *hlb* (Egyir et al., 2024; Lebughe et al., 2017; Shittu et al., 2021). The co-occurrence of PVL and β-hemolysin may enhance pathogenicity, as Hlb-sphingomyelinase activity can synergize with PVL-mediated leukocyte cytotoxicity, potentially promoting tissue colonization, destruction, and immune evasion (Di Bella et al., 2025).

Our study found diverse *S. aureus* lineages in some clinical presentations (Table 4). We observed diverse *S. aureus* lineages (>4 CCs) in bloodstream, upper respiratory tract, urogenital, and wound samples. *S. aureus* strains assigned to CC1 and ST152 were identified in all categories of clinical presentations, whereas those grouped with CC8 were observed in all infection groups except eye infections. Moreover, phylogenetic analysis revealed strain diversity across both inpatient and outpatient units (Figure 1), suggesting overlapping transmission dynamics among lineages, particularly classical community-associated MRSA (CA-MRSA) clones. SCC*mec* types IV and V are classically linked to CA-MRSA (Lakhundi and Zhang, 2018). The substantial genetic heterogeneity suggests possible endemic circulation of different lineages in the healthcare institution. The emergence of PVL-positive clones (e.g., ST152-MRSA-V and CC1-ST772-MRSA-V) across different wards, particularly in the NICU, raises the possibility of localized nosocomial transmission. These findings underscore the importance of molecular surveillance, improved infection prevention and control strategies, and integrating genomic data with clinical information to mitigate MRSA spread in hospital settings.

This study has some limitations. The retrospective, single-centre design carries inherent selection bias, and the disproportionate representation of wound infection strains (52%) might limit the generalization of the findings to broader clinical and community settings. Nevertheless, this study provides a comprehensive genomic study of *S. aureus* in North Central Nigeria. It highlights the emergence of MDR and hypervirulent *S. aureus* clones in the health-care facility. Although the number of strains analyzed is insufficient to generalize the findings, identifying hypervirulent and MDR *S. aureus* clones in the hospital underscores the need for effective infection prevention and control strategies, antimicrobial stewardship, and genomic surveillance.

## 5 Conclusion

This study describes a diverse *S. aureus* clonal population and high prevalence of MRSA and MDR *S. aureus* in a tertiary hospital in North Central Nigeria. The emergence of hypervirulent MRSA lineages, particularly CC1-ST772 and ST152, poses significant challenges for clinical management and infection control. These findings underscore the need for genomic surveillance integrated with infection prevention and control and antimicrobial stewardship programs to limit the spread of these high-risk clones.

## Data Availability

The datasets presented in this study can be found in online repositories. The names of the repository/repositories and accession number(s) can be found in the article/Supplementary material.

## Footnotes

1
https://cge.cbs.dtu.dk/services/SCC*mec*Finder/

2
https://usegalaxy.org
